# 

**Published:** 1917-09-15

**Authors:** 


					Public Authorities as Champions of
Individuals.
The Local Government Board for Ireland issued
a recommendation to local boards of guardians to
elect as medical officers to appointments falling
vacant men who were unfit for military service.
The Guardians of Bal.rothery Union appointed a
medical officer who was of military age and, on the
Local Government Board declining to recognise the
appointment, the Union mentioned commenced pro-
ceedings to compel the central authority to sanction
it. When the case was about to be heard certifi-
cates of unfitness granted to the doctor in question
were produced, the effect of which on the action was
virtually to leave no question in dispute. On appli-
cation being made to take the case out of the list,
Mr. Justice Gibson ordered each party to pay their
own costs,' and he remarked that the case was an
illustration of the inconvenience and unwisdom of
public authorities taking up the cases of individuals,
instead of leaving them to establish their own
rights.
Training for Welfare Supervisors.
A special course of training for Welfare Supervisors
is being arranged by the Eatan Tata Department of Social
Science and Administration, University of London, with
the assistance of the Health and Welfare Section of the
Ministry of Munitions. The course will be held at the
London School of Economics and Political Science, and
will extend over nine months (October to June). It will
include both theoretical instruction and practical work.
498 THE HOSPITAL September 15, 1917.
Matrons' and Sisters'
Appointments.
Colony for Epileptics, Langho, near Blackburn.?Miss
K. E. Oyler has been appointed, matron. She was trained
at Guy's Hospital, and has been matron at the Royal
Infirmary, Gloucester.
Felixstowe Cottage Hospital.?Nurse Kathleen Boatty,
who was trained at the London Hospital, has been ap-
pointed matron.
Hayes Cottage Hospital, Middlesex.?Miss Maudo Haines
has been appointed matron. She was trained at Fulham
Infirmary, and has worked under the Joint War Committee
since 1915. She holds the certificate of the Central Mid-
wives Board. /
Royal Victoria and West Hants Hospital, Bournemouth
(Boscombe Branch).?Miss Elsie G. Gale has been appointed
matron.' She was trained at the General Hospital, Chelten-
ham ; has been night superintendent at the Chester Royal
Infirmary; assistant matron at the York County Hospital
and at the West London Hospital.
Skipton-in-Craven Joint Infectious Diseases Hospital.?
Miss Frances Pearson has been appointed matron. She
was trained at the Bethnal Green Infirmary and the
Huddersfield Sanatorium, since serving as sister at Keighley
Isolation Hospital, as home sister and assistant matron at
Bradford City Hospital, as assistant superintendent at
Liverpool Convalescent Institution, and as matron at Brad-
well Joint Hospital, Chesterton.
Southwold Cottage Hospital.?Miss F. M. Austen has
been appointed matron. She was trained at Charing Cross
Hospital and the Chelsea Hospital for Women, and has
eince been matron of the Faringdon Cottage Hospital.
Mansfield and District Hospital.?Miss D. F. Cocking
has been appointed massage and electrical sister. She
was trained at Manchester Royal Infirmary, where she
was later, sister. She has since been sister at the Royal
Sea Bathing Hospital, Margate, and massage and elec-
trical sister at Lady George Nevill's Hospital, Hove.
Town Close Lodge V.A.D. Hospital, Norwich.?Mrs.
Evelyn A. Burling has been appointed night sister in
charge. She was trained at the Bristol General Hospital,
and has been staff nurse at cue West London Hospital,
staff -nurse and acting night sister at tho National Hospital
for the Paralysed and Epileptic, sister at the Dulwich In-
firmary, and special staff nurse at St. Bartholmew's Hos-
pital.
Hospital of St. Cross, Rugby.?Miss Caroline Wilson has
been appointed sister. She was trained at the Royal
Hospital for Sick Children, Aberdeen; has been sister at
the Hospital for Women and Children, Edinburgh, and at
the Red Cross Hospital, Newnham Paddox, Lutterworth.
NoTE,?Particulars of Appointments for Insertion In this
column will be welcomed.
Personalia.
Capt. E. Talbot, formerly senior house surgeon at
Manchester Infirmary, has been awarded the Military
Cross. \
Mr. W. S. Wintle, the secretary of the Foundling
Hospital, is retiring after fifty-one years' service of
arduous and devoted work for the institution.
Temp. Lieut.-Col. T. P. Legg, M.S., M.B., F.R.C.S.,
who has received the C.M.G. for services in Mesopotamia,
has been house surgeon at St. Bartholomew's and at the
Royal Free Hospitals. He was subsequently appointed
registrar at King's College Hospital, where he is one of
the visiting surgeons. ? Some two years ago, by invitation
of the War Office, he undertook hospital work at Malta,
then in Sicily, afterwards proceeding to Mesopotamia.
Cambridge and District Workers'
Hospital Fund.
The seventh annual meeting of the Cambridge and Dis-
trict Workers' Hospital Fund was held in the lecture-room
at Addenbrooke's Hospital on August 29. In the absence
of the chairman (Mr. S. Gymer), Mr. A. T. Potter, vice-
chairman of the Workers' Hospital Fund Committee, pre-
sided.
What can be Accomplished by the Penny.
During the seven years the Workers' Hospital Fund has
been in existence it has collected 950,000 pennies, and
has distributed nearly ?4,000, of which over ?3,000 has
gono to Addenbrooke's Hospital, and nearly ?400 to the
Cambridge District Nursing Association. The Fund has
a Convalescent Department, which is of great value to
the workers in time of exceptional'need.
It was reported that, in spite of the fact that so many
of the original contributors were now serving their King
and country, contributions had been received from no
fewer than eighty centres, whilst the income for the year
(excluding bank interest, ?4 8s.) amounted to ?464 4s. 4d.,
as compared with ?476 19s. in the previous year. The
expenditure for the year amounted,to ?9 18s. 6d. The
balance of ?458 13s. 8d. was allocated as follows : Adden-
brooke's Hospital, seven-tenths, as per rules, ?321; Cam-
bridge District Nursing Association, ?30; New Cherry-
hinton Nursing Association, ?8; Royal Surgical Aid
Association, ?7; honorarium to secretary, ?7 7s.; total,
?373 7s.; Addenbrooke's Hospital additional grant, ?65;
transferred to Convalescent Fund, ?20 6s. 8d; grand total,
?458 13s. 8d.
Colonel Harding, the president of the Fund, expressed
his admiration of the untiring zeal and great devo-
tion of the members and contributors in raising
year by year such a substantial sum. He re-
ferred to the great difficulties under which the work of
Addenbrooke's had been carried on under war conditions,
and paid tribute to the work of all connected with the
institution, including the governors and every member of
the staff. In receiving the cheque for ?386 for Adden-
brooke's, Colonel Harding thanked all the workers from the
bottom of his heart for their generous contribution, ex-
pressing his deep appreciation of the work they had been
doing and congratulating them upon the success which had
attended their efforts.
Gratitude was similarly expressed by Mrs. Steel and
Mrs. Clark on behalf of the Cambridge District Nursing
Association and the New Cherryhinton District Nursing
Association respectively.
Sympathy and Brotherhood.
The Secretary-Superintendent, in congratulating and
thanking the members of the Workers' Fund, emphasised
the necessity of proving themselves worthy of the great
sacrifices being made by everyone, and referred to the
striking indication of unmistakable Christian sympathy
and brotherhood which he had observed whilst travelling
about and addressing meetings on the work and needs
of the hospital.
The Chairman took the opportunity of thanking the
matron, sisters, and nurses for what they had done for
members of the Fund, and, in reply, Miss Crockenden
said how glad they were to have an opportunity of show-
ing their gratitude for the workers' extraordinary gener-
osity to Addenbrooke's year after year.
Mr. A. T. Potter was elected chairman for the coming
year; Mr. E. J. Marchant, vice-chairman; Mr. E. J-
Papworth, secretary; and Mr. F. Cant, treasurer.
Manager's Notices.
Now Ready, "The Hospital" Index to Vol. LXI., Oct. 1916 to April 1917. Price 2?d. per copy post frea
All letters on business matters must be addressed
to The Manager, " The Hospital," 28 and 29 8outh*
ampton Street, London, W.C. 2.
Subscriptions, which may commence at any time, are
payable in advance. Cheques and Post-Office Orders
should be crossed London County and Westminster Bank,
Covent Garden Branch, and made payable to the Manager
of The Hospital. n
Rates of Subscription (payable in advance).
UNITED KINGDOM).
Three Months (including Postage)  2?. 04
Six Months do.  4s. Od
Twelve Months do.  6s. 6d.
FOREIGN AND COLONIAL.
Three M'onths (including Postage)  2s. 6d.
Six Months do  5s. Od.
Twelve Months do.  8s. 8d.

				

